# Surgical Observations, Part IV

**Published:** 1818-03

**Authors:** 


*Surgical Observations, Part IV.* By Charles Bell.

This Part contains Report of Cases of the Acute Fungus Tumour, or soft Cancer; Report of Cases of Tumours which take their Rise from the Gums and Alveoli; Report of Gun-shot Wounds of the Knee Joints; Report on Sacs formed in the Urinary Organs; Report on Fracture of the Skull, &c. all illustrated by Cases, with five plates.

1. *Soft Cancer.* This is the most terrible tumour to which the human body is subject; for, long before danger is indicated, the patient is beyond the power of remedy. When amputation is deemed advisable, the prognosis is often uncertain; but the diagnosis is nevertheless very desirable.

"It is very important to know the early character of the tumour; and I think the authors who have treated of this disease, have dwelt chiefly on its appearance in the latter stage. In the beginning, and whilst the tumour is deep, I know not how its nature can be determined; but when it becomes prominent, it is peculiarly tense and elastic. In its progress it is not uniform; but having projected in a small hemisphere, another of corresponding form and dimensions rises beside the first: in succession another lobe or division is produced; and so it proceeds until the tumour has assumed a very formidable magnitude. The last formed knob always is more full and elastic, with a fresher colour and more vascularity. Those formed earlier become somewhat shrivelled, and present to the finger more the feeling of a solid, the surface being firmer and irregular. There is no pain for a long time, unless the place of the tumour subject it to the action of the muscles, or press it against the nerves, or impede the motion of a joint. In some of these cases, however, the pain of the tumour in the latter period was so great, that the suffering of amputation was considered as the lesser evil. As the tumour enlarges, the cutaneous veins become more conspicuous, and on the convexity of the more elastic projections the smaller vessels are numerous. The colour of such projecting parts of the tumour is peculiar. There is a faint yellowish red, deepened by the ramifications of these cutaneous vessels.

"On the prominence of some of the older tubercles the cuticle breaks; a pale fluid exudes from small holes. From the same source there in time springs up a spongy and luxuriant fungus. This fungus frequently bleeds; and it is this character which induced Mr. Hey to give the disease the name of fungus hæmatodes. By this time the disease is propagated to other parts of the system; to some of the viscera of the abdomen, and most commonly to the liver; and there are felt internal pains, which, although they may be relieved by bleeding, and blisters, are fatal signs. For now we observe a rapid and singular termination of the disease in death. The constitutional powers are suddenly affected; the patient is seized with rigors, attended with sickness; the countenance suddenly changes; it becomes pale, and of dingy hue or earthy colour. Sometimes the skin assumes a very peculiar bright yellowish tinge. In the mean time there is a remarkable irritability of stomach, so that nothing will stay on it; the pulse becomes very feeble and rapid; the patient has internal pains; he becomes more and more feeble, insensible, and dies. From the occurrence of these constitutional symptoms, the patient dies in the space of from three to eight days." P. 371.

*Case.* Robert Clark, ætat. 68, brought to the hospital, June 1, 1815, for a large tumour seated in the groin, which he thinks originated in a strain. It is now the size of a melon; embraces the crural artery and vein; colour dark purple; surface irregular; resistance unequal. Complains of tension and numbness in the thigh. — June 6th. The disease pronounced fungus hæmatodes. The colour is darker, with tingling pain. — 17th. The tumour is increasing, with œdema of the leg. Died on the 8th of July. On dissection, the tumour was found to consist of a greyish mass, streaked with blood. All the lumbar glands partook of the disease. The coats of the crural artery were soft, and participated in the disease.

We shall pass over several interesting cases, in order to delineate one which has been furnished by that accurate observer, and enlightened physician, Dr. Dickson, of Clifton.

Robert Lane, of a scrophulous habit, and drunken character, had his left leg jammed in the main hatchway, but continued at his duty for ten or fourteen days. Then came on pain, increased by exercise, which continued for a month to augment. The integuments about the head of the fibula inflamed; the swelling was tense and shining. The accident happened on the 5th of May, and on the 25th of July he was received into the naval hospital. In the end of September, the tumour measured 23 inches in circumference, occupying the upper part of the leg; unequally elastic, with many conspicuous veins on the surface. — Sept. 28. It was punctured, when a stream of pure venous blood flowed out, requiring compress and bandage to restrain it. After this, a tenacious sizy fluid oozed out in great quantity. On the 2d of October, the tumour presented a livid appearance, with a substance protruding from the puncture-wound. A probe could now be passed in all directions through the wound. On the 5th, the swelling extended above the knee, and inflamed lymphatics could be traced along the thigh. The tumour and fungous protrusion now rapidly increased, attended with night sweats, small, quick pulse, and flushed face.—Amputation. The tumour presented a mass like brain in consistence and colour. Fibula carious nearly throughout; tibia diseased; arteries were entire, but ossified. Various ossific depositions throughout the tumour. The patient did well till the 5th day, when, on the first dressing, the integuments were found partially adherent, with pus covering the wound. On the 6th, a hæmorrhage. 7th. Profuse discharge of thin matter, and now a tumour appeared in the middle of the stump; another hæmorrhage. 8th. Hæmorrhage profuse; pulse quick, small, hurried; countenance altered. Finally, on the 11th, tetanus appeared, and on the same day he died.

On examining the stump, the periosteum had separated from the bone, and the tumour was seen to arise from the medulla of the bone. It cut like the cortical part of the brain.

The 8th case is singular, and we shall give it entire.

"About three years before I saw the subject of the following Case, he had fallen from the side of a ship. It happened in this way: Seeing his fellow workman falling, he threw himself forward to break his fall, and succeeded; but in doing this, he fell himself; for he was caught by the ham, on a projecting bolt in the side of the ship, over which he turned and hung suspended. He suffered much from the bruise on the back of the thigh, but in a short time got entirely well.

"Some time after this, he began to be much troubled with a pain in his foot. This pain was in a part not likely to procure him much sympathy; and he suffered much and long, without attempting to procure assistance, or only such as the extremity of pain would induce a man to try.

"But the pain continued to increase from day to day, until it totally unfitted him for labour, exhausting and wasting his frame by continued watching. This pain was of a peculiar kind; it was confined to the bottom of the foot, and was like an intense burning, while there was not the slightest discolouration or swelling in the place. Often he would rise at night from his bed, and stand on the cold stones, or plunge his foot into warm water or cold water, or alternately.

"He now sought relief in a public hospital, and the attendants, disconcerted with the strangeness of the symptoms, which they did not comprehend, put him, as is usual on such occasions, on a course of mercury; but this trial of a medicine did no good, and he went home. But still suffering continually, he was induced, after a lapse of some months, to return to the hospital, and was again put under a more severe and a longer continued course of mercury than before. By the time this was over, he had suffered continually for two years, and was reduced to a skeleton, and was far gone in hectic.

"When I saw him, he gave me this account, and then continued to complain of the extreme pain in the sole of the foot. He told me too, that he had a strange numbness of the leg when he sat down. On examining into this circumstance, which I thought would lead to some explanation of the more prominent symptom, I found a tumour in the ham, which, when pressed, gave no particular pain, but rather a sense of pricking numbness down the leg. The tumour was to the feeling of a bony hardness. I conjectured that there was some tumour pressing and wedging upon the popliteal nerve; and that this injury to the nerve in its course was referable, by the patient's feelings, to the extremity and final distribution of the nerve. I thought of an operation, yet I was deterred from it by the dying state of the poor man, who now suffered but indirectly from the disease of the leg, and in all probability death was no longer to be avoided by the removal of the original cause. I thought that he might be brought round, so as to gain some strength; but within the week he died."

*Dissection.* "The examination of the cavities was not permitted. On dissecting the limb, I found a tumour under the fascia, and about three inches higher than the usual place of popliteal aneurism. I found some nerves running over it, of a remarkably pure whiteness. On tracing the sacro-ischiatic nerve, I found it enter into the substance of the tumour; but on more careful observation, I found that the peroneal or fibular nerve, though close on the tumour, was not incorporated with it; but that the tibial nerve was incorporated with the tumour. On making the section of the tumour, it had much the appearance of a large ganglion on the tibial nerve: the fasciculi of the nerve could be traced only a little way into its substance; and in the interstices of the fasciculated bands, a vascular fatty substance could be observed, which resembled marrow. By more experience, I now recognized in this matter the distinguishing character of the cancerous tumour we are now considering.

"I think it is impossible to mistake the nature of the symptoms. I have no doubt that the injury received on the ham was the cause of the disease in the nerve, as in other cases we have seen a blow produce the disease in the bone: yet I think we cannot close our eyes to the striking proof of the affection of a nerve in its course being referable to its extremity. Had the nature of this disease been understood earlier, I have little doubt that cutting across the portion of the popliteal nerve, which forms the tibial nerve, and the extirpation of the tumour, would have been succeeded by perfect relief from pain, at the expence of losing the use of the leg." P. 405.

The Report is wound up by some pertinent observations on the structure of soft cancer, for which we must refer to the work itself.

Report II. *Tumours arising from the Gums and Alveoli.*

Small tumours in these situations often remain stationary for a long time, but at length force their way into the bones of the face, filling up the cells and the cavities of the nose, pressing out the eyes, and rising at last upon the basis of the brain itself. Thus it occupies the whole face, extinguishing in succession the organs of the senses; impedes utterance, and finally destroys the patient by pressure on the brain. Mr. B. suspects that the teeth, the alveoli, aud the gums, have something in common, or, as it were, the same constitution. This suggests to us, that when the disease originates in the gum, it may belong to the tooth and alveolar process also. On this process of reasoning, Mr. B. has been induced to cut away gum, alveolar process, and tooth, along with the tumour that springs from the gums.

"This tumour first shews itself in a small hard prominence of the gum, shooting out betwixt two of the teeth; and the teeth being good, is an unfavourable circumstance, for when they have become loose, and are displaced, without being themselves diseased, it implies that the cause is deep, and not to be removed by pulling the teeth. If the teeth be carious, and originally faulty, we have a reasonable expectation of arresting the progress of the disease, by removing the teeth; but when, independent of the teeth, the tumour has its origin in the membrane of the fang, or in the socket, we cannot hope to extirpate the disease, without removing the whole system of parts, the whole of what is connected in constitution." P. 416.

The first Case led to a bold operation, which does great credit to Mr. Bell's courage and skill; it was, however, unsuccessful. The second Case was more fortunate, and it we shall condense.

September 6th. —— Burrows. One side of the upper jaw is occupied by a tumour which has displaced the teeth, and projects into the mouth. The tumour is firm, and has little sensibility, bearing the impression of the lower jaw teeth.—Mr. B. first removed the teeth, then divided the gum and alveolar process across with a saw, to the depth of three quarters of an inch anterior to the tumour. He next cut across the alveolar circle behind the tumour. An incision was then made with the scalpel, which cut the tumour from the cheek; another was made in the angle betwixt the projecting tumour and the roof of the mouth. A small saw was now introduced betwixt the tumour and the cheek, and the jaw-bone cut across above the range of the fangs of the teeth. The whole was then torn away with large forceps. A stream of blood jetted from the jaw, supposed to come from the enlarged alveolar artery. A piece of cork, fitted to occupy the chasm, was introduced on some lint dipped in tinct. ferri mur. The patient was then made to close her teeth upon the cork, and the hæmorrhage was suppressed. This woman recovered, and, twelve months after the operation, continued well. But this period, Mr. B. justly observes, is no security against a return of this formidable disease.

REPORT III. *On Gun-shot Wounds of the Knee-Joint.*

Our author makes a few general reflections on this subject, and then minutely details a very interesting case, of which we shall presently give an outline. He observes, that *very few* histories of gun-shot wounds are given in detail. This is rather a sweeping expression, and we think we could point out such a number as would overthrow the position; but we are not, at present, in the humour for cavilling about expressions.

When a musket-ball is lost in the fleshy substance of the body, modern surgery forbids our making much search for it, by "poking with the probe," or many free incisions. But it is questionable if this rule holds good in regard to the wounds of joints and the heads of bones engaged in the articulations. In a joint, where the texture is peculiar, and the parts constantly in motion, the presence of a ball causes great inflammation, protracted suffering, and lameness. If possible, therefore, the ball should be discovered and extracted previously to the commencement of re-action in the parts. Here every thing depends on the judgment of the surgeon, since the degree of injury, which the constitution will safely bear, must be calculated. An absolute rule is therefore just as likely to lead to error, as to direct the practice aright.

*Case of Baron Dreissen, a Russian General.* This officer was wounded in the memorable battle of Borodino, in September 1812. A musket ball was supposed to strike him in the most prominent part of the inner condyle of the left femur. It was treated as trifling on the field of battle; but when carried to Moscow, the patient's sufferings were extreme. The wound closed, and the knee swelled prodigiously. Cataplasms re-opened the wound, and brought on a discharge. He was now carried to the town of Murom, where Muchin, a famous Russian operator, examined him; but as eight days had now elapsed, the track of the ball could not be ascertained. In January 1813, at Moscow, Muchin thought he could feel the ball, and attempted an extraction; but the ball was immoveable, and the surgeon desisted, and used injections of myrrh and bark, with compression, to close various sinuses that had formed. Nothing at last remained but the canal of the original wound and the ball at the bottom of it. In July, the General was so much better as to be able to travel to Petersburgh, where Mr. Bush, Professor of the academy, made many attempts to extract the ball, but all in vain. He advised him to trust to nature and time. In the mean time, another surgeon made trial of the spunge tent, which brought on violent tensive inflammation, by which the sponge was jammed in the wound, and obliged to be dragged away by force. In February 1814, the General managed to join the head quarters of the Emperor at Troyes. Here our countryman, Sir James Wylie, hesitated between amputation and the trepan, but in the critical state of affairs, delivered the patient over to the English surgeons.

On the Baron's arrival in London, Mr. Bell found his health much broken down by the long continued irritation of the wound. His pulse was quick; he had frequent febrile paroxysms, ushered in by rigors. The wound was so irritable that the gentlest introduction of the probe brought on an attack of fever. He was removed into the country, and Mr. B. very honourably desired a consultation. When the Baron had so far recovered as to be able to bear an examination, it was found that the probe introduced into the wound passed three inches and a half before the rub of the ball could be felt. The probe passed a little obliquely downwards and backwards through the substance of the head of the femur. The ball appeared to be lodged in the bone. An attempt was made, by the advice of Sir E. Home, to enlarge the wound, by the use of the lapis infernalis; the consequence was, a severe attack of erysipelas, which extended from the groin to the toe. After recovering from this, Mr. B. made various attempts to extract the ball; but owing to its great depth in solid bone, the sensibility of the parts, and the constitutional irritability, he was forced to desist. After this, it was determined to let the wound close, and the Baron returned to the continent. It continued closed all the year 1815, and till March 1816, when it opened, preceded by great swelling and inflammation of sixteen days' continuance. An abscess was at the same time formed on the outside of the knee, which was opened by M. Gesling, Surgeon of the Imperial Guard, and vent given to a great deal of water. The Baron having in some degree recovered, Surgeon Krestowsky attempted to dissolve the ball by pouring quicksilver into the wound. In September 1816, the Baron returned to London, when he shewed Mr. B. a quantity of quicksilver, which had, at different times, issued from the sinus on the outside of the knee-joint. Mr. B. thinks the presence of the quicksilver occasioned more distress, swelling, and discharge than would otherwise have taken place.

In the month of December the Baron began to suffer in an extraordinary manner. Violent paroxysms of pain came on suddenly, and continued for one or two nights, after which they subsided: these returned again and again with increased violence. The excess of pain was such as, at one time, to deprive him of his senses for a quarter of an hour. It appeared that the fibular nerve was involved in the inflammation, as a burning sensation shot to his foot, along the whole course of the nerve. The sinus was now enlarged, and matter mixed with quicksilver discharged, which, for a time, relieved him.

In a consultation, however, it was recommended to desist for a time from all operative measures. The rapid returns of pain, with fever and nervous retchings, determined Mr. Bell to amputate.

*Dissection.* Mr. B. enlarged the sinus on the outside of the knee, so as to admit the finger betwixt the popliteal vessels and the lower head of the femur; but no indication of the presence of the ball was to be felt. In prosecuting the dissection, a great quantity of mercury was found lodged in different abscesses around the joint. There was an abscess between the heads of the gastrocnemius muscle, and a sinus ran high up along the side of the popliteal vessels. There was a diseased portion resembling fungus, which surrounded the fibular nerve, and in which was contained innumerable small globules of mercury. The trunk of the popliteal nerve was surrounded with a diseased portion of the same kind, in which were also globules of quicksilver. On calculating the level of the ball, the femur was cut so as to disclose it. It lay in the centre of the outer condyle, surrounded by a layer of soft membrane resembling red velvet. It was, notwithstanding, firmly impacted, and although one half of it was exposed, it stuck firmly There was a small cavity in the bone behind the ball, which communicated with the joint and with the sinuses. The patella, tibia, and fibula, were osseously united. The periosteum covering the head of the femur was inflamed, and the surrounding parts thickened and diseased. The popliteal vessels and nerve were, through their whole extent, involved in condensed cellular membrane, making a firm mass.

An interval of ease succeeded the amputation; but bye and bye the strokes of pain were renewed, and referred to the same part of the dorsum of the foot as formerly, and attended with the exact same contractions of the toes, (in imagination) and all other morbid sensations. The thigh swelled several times, and depots of matter formed in various parts, requiring artificial vents. At length, however, this unfortunate officer recovered so far as to be able to embark for his own country on the 3d of May 1817.

The next Report, on the formation of sacs in the urinary bladder, we must pass over unnoticed, because Mr. Bell's observations have, in general, reference to preparations in Windmill Street; and, of course, are of less utility to those beyond the circle of the metropolis. But the

Report on fractures and counter-fissures, &c. of the skull is too important to be slightly analysed.

Mr. B. commences this Report with some judicious advice to the young surgeon, on his being called to an accident. If a dislocation or fracture, before we seize on the limb, or tear off the dressings, we should sit down calmly and examine into the circumstances attendant on the injury, by which investigation much knowledge may be gained.

Mr. Bell here takes occasion to question the accuracy of some of Mr. Abernethy's opinions respecting the effusion of blood on the dura mater from rupture of the middle artery of that covering. Mr. B. thinks that the power of the artery is inadequate to the effect of separating the dura mater from the skull; but that, on the contrary, this membrane is loosened by the blow, and the sanguineous effusion is the *effect*, not the cause of the separation. There is certainly much plausibility in Mr. B's reasoning; yet, when we consider that the whole force of the arterial system immediately acts on the ruptured point of an artery; and when we reflect on the tensive injections of limbs resulting from punctured blood-vessels, we shall probably pause ere we hastily reject the opinion of Mr. Abernethy.—Mr. B. illustrates his position thus:

"Strike the skull of the subject with a heavy mallet: on dissecting, you find the dura mater to be shaken from the skull at the part struck. Repeat the experiment on another subject, and inject the head minutely with size injection, and you will find a clot of the injection lying betwixt the skull and the dura mater, at the part struck, and having an exact resemblance to the coagulum found after violent blows on the head. This proves also that the advice is wrong which would hurry the young surgeon to trepan the skull immediately, to preserve the life of the patient, and to prevent the hæmorrhage from being profuse: for this idea is given on the supposition that the artery is slowly separating the membrane from the bone by the force of arterial pulsation." P. 467.

*Case.* April 16th. A man was brought to the hospital insensible from a blow on the head from the fall of a bucket from a great height. Pulse 56; breathing slow; pupils contracted, previously dilated. Considerable tumefaction of the scalp behind the ear, and above the transverse line of the occipital bone. Draws a deep sigh at short intervals, with snorting respiration in the intervals. Pulse irregular. Scalp opened, but no fissure or depression observable. Six ounces of blood drawn from the occipital artery, and thirty from the arm. The pulse increased in quickness after the last bleeding. He died on the second day.

*Dissection.* Coagulated blood lay under the skull, and extended over all the basis cranii. No fissure in the skull-cap, but a rent was discovered in the base of the skull. It began by the side of the petrous bone of one side, and extended round the occipital bone behind the foramen magnum to the petrous portion of the temporal bone on the opposite side. The blow had been received on the strongest part of the skull, the fissure was in the weakest.

*Case.* A young man fell from the parapet of a wall, and pitched with his head on the area, a height of five feet. Brought into the hospital stunned; but recovered as from a slight concussion, and by the usual means apparently got well. He had been dismissed, and gone into the board-room to return thanks, but suddenly fell down and died.

*Dissection.* The margin of the foramen magnum was fractured, and it appeared, that on suddenly turning round the head, the condyle was displaced, and the loose bone brought to press and nip the medulla oblongata: in short, the poor fellow was pithed.

*Case.* A man fell from a height and injured the right parietal bone, near the coronal suture. He recovered from the effects of concussion; but after an interval he was attacked with rigors, became quite insensible, and lay with stertorous breathing and a total relaxation of one side. The bone was exposed and examined: it was found bare, the pericranium having separated from its surface. The trephine was applied, but no matter found. The patient died next day.

*Dissection.* The left posterior lobe of the cerebrum was found inflamed, and covered with purulent matter.

Our limits compel us to close our analysis. Mr. B. has here concluded the first volume of his Reports, and hints that we are not to expect regularity in the appearance of the succeeding parts. We are sorry for this: for although order and composition were not strictly attended to, and we could not say—

Materiam superabat opus;

Yet the value of the *matter* made ample amends for the defect of the *manner*. We trust, therefore, that he does not begin to *flag;* and that we shall not have occasion to apply the vulgar adage of the "*new broom,*" to the Clinical Reporter of Middlesex Hospital.

